# Highly Effective Renaturation of a Streptokinase from *Streptococcus pyogenes* DT7 as Inclusion Bodies Overexpressed in *Escherichia coli*

**DOI:** 10.1155/2014/324705

**Published:** 2014-05-05

**Authors:** Sy Le Thanh Nguyen, Dinh Thi Quyen, Hong Diep Vu

**Affiliations:** ^1^Institute of Biotechnology, Vietnam Academy of Science and Technology, 18 Hoang Quoc Viet Road, District of Cau Giay, Hanoi 10600, Vietnam; ^2^Department of Biotechnology and Pharmacology, University of Science and Technology of Hanoi, 18 Hoang Quoc Viet Road, District of Cau Giay, Hanoi 10600, Vietnam

## Abstract

The streptokinase (SK) is emerging as an important thrombolytic therapy agent in the treatment of patients suffering from cardiovascular diseases. We reported highly effective renaturation of a SK from *S. pyogeness* DT7 overexpressed in *E. coli*, purification, and biochemical characterization. A gene coding for the SK was cloned from *S. pyogeness* DT7. Because accumulation of active SK is toxic to the host cells, we have expressed it in the form of inclusion bodies. The mature protein was overexpressed in *E. coli* BL21 DE3/pESK under the control of the strong promoter *tac* induced by IPTG with a level of 60% of the total cell proteins. The activity of the rSK, renatured in phosphate buffer supplemented with Triton X-100 and glycerol, was covered with up to 41 folds of its initial activity. The purified of protein was identified with MALDI-TOF mass spectrometry through four peptide fragments, which showed 100% identification to the corresponding peptides of the putative SK from GenBank. Due to overexpression and highly effective renaturation of large amounts of inclusion bodies, the recombinant *E. coli* BL21 DE3/pESK system could be potentially applied for large-scale production of SK used in the therapy of acute myocardial infarction.

## 1. Introduction

Streptokinase (EC 3.4.99.22) (SK), a commercially important nonprotease, binds stoichiometrically to both circulating and thrombus-bound plasminogen (Plg) to generate SK-plasminogen activator complex. Cleavage of plasminogen in zymogen form at an Arg-Val bond generates plasmin, an active enzyme that degrades fibrin component of thrombin [1]. Due to this property, the streptokinase has been widely used in the therapy of acute myocardial infarction for its strong activity in dissolving blood clots [2].

Most group A, C, and G *β*-hemolytic streptococci isolated from human hosts secrete streptokinase with molecular mass of 47 kDa, which convert the plasminogen to the serine protease plasmin. However, due to low SK production yields from natural host and its pathogenicity, so research interest has shifted to cloning and expression of SK in hyperproductive and safe heterologous host systems. Therefore,* sk* genes have been cloned and expressed in different expression systems including* Bacillus subtilis *[3],* Streptococcus sanguis* [4],* Streptomyces lividans* [5, 6],* Schizosaccharomyces pombe *[7],* Pichia pastoris *[1, 8],* Lactococcus lactis *[9], and* Escherichia coli *[10, 11]. However, there are some disadvantages of producing recombinant proteins in* Pichia pastoris *due to high glycosylation level [12] or in* Lactococcus lactis* due to low cell density [9].

*Escherichia coli* is the most commonly used host for the production of recombinant proteins, both in research and industry [13]. High-level expression of recombinant proteins in the form of a soluble intracellular product, secretory product, or as insoluble inclusion bodies depends on promoter system, host-vector interactions, sequence, and characteristics of recombinant products and the effect of the expressed foreign protein on host cell physiology [14].

The expression of SK as inclusion bodies by* E. coli* systems is shown to be useful for obtaining large amounts of protein, provided that renaturation is effective and recovery of active protein is high. Thus, the purpose of this study was firstly to overproduce the recombinant streptokinase in* E. coli* BL21 (DE3) and simultaneously to refold effectively the large amount of the recombinant streptokinase as inclusion bodies overexpressed by* E. coli* BL21 (DE3) under the control of the promoter T7. Only both objectives were gained; then the recombinant* E. coli* overproducing SK as inclusion bodies can become a potential strain for industrial SK production.

## 2. Materials and Methods

### 2.1. Chemicals and Reagents

DNA cloning kit, RNase A, restriction enzymes (*Bam*HI,* Not*I, and* Eco*RI), T4-ligase, and Proteinase K were purchased from Fermentas (Thermo Fisher Scientific Inc., Waltham, USA). The DNA Extraction Kit was from Qiagen (Venlo, Netherlands). Protein Extraction Kit and ProBond resin were supplied by Invitrogen Corp. (Carlsbad, CA, USA). Human plasminogen from MP Biomedicals (Santa Ana, USA); SK, N (p-tosyl) gly-pro-lys-4-nitro anilide acetate salt (AAS), SDS from Sigma Aldrich Co. (St. Luis, USA); Plasminogen, Tween 20 and Tween 80 from BioBasic Inc. (NY, USA); Triton X-100 and EDTA from Merck (Darmstadt, Germany). All other reagents were of analytical grade unless otherwise stated.

### 2.2. Plasmids, Bacterial Strains, and Culture Conditions

The bacterial strain* Streptococcus pyogenes* DT7 (GQ247718) isolated from a patient at the Army Hospital No. 103 (Hanoi, Vietnam) was used as the source of the streptokinase (*sk*) gene.* Escherichia coli* DH5*α* (F^−^, ø80d*lacZ*ΔM15, Δ(*lacZYA-argF*) U169,* deo*R,* recA*1,* endA*1,* hsdR*17(rK^−^, mK^+^),* phoA*,* supE*44, *λ*–,* thi*-1,* gyrA*96,* relA*1) and the vector pJET1.2/blunt (Fermentas, Thermo Fisher Scientific Inc., Waltham, USA) were used for DNA manipulations and amplification.* Escherichia coli* BL21 (DE3) cells (*F* ^*–*^ *ompT gal dcm lon hsdS*_*B*_ (*r*_*B*_^−^ *m*_*B*_^−^) **λ**(*DE3* [*lacI lacUV5-T7 gene 1 ind1 sam7 nin5*]) and pET22b+ vector (Novagen, Merck KGaA, Darmstadt, Germany) were used for expression of SK. LB medium (Luria-Bertani) containing 1% (w/v) bacto tryptone; 0.5% (w/v) yeast extract; 1% (w/v) NaCl; pH 7–7.5 was used for cultivation of* E. coli* DH5*α* and BL21 (DE3). LB agar contained additionally 2% (w/v) agar and 100 *μ*g ampicillin/mL.

### 2.3. DNA Manipulations

Genomic and plasmid DNA isolation was carried out by methods which have been previously described [15]. DNA fragments and PCR products were excised from a 0.8% agarose gel and purified by a gel extraction kit (Qiagen, Venlo, The Netherlands) according to the manufacturer's instructions. DNA sequencing was performed on an ABI PRISM 3100 Avant Genetic Analyzer (Applied Biosystems Inc., Foster City, USA).* E. coli* DH5*α* and BL21 were transformed using heat shock method that has been previously described [15].

### 2.4. DNA Amplification and Plasmid Construction

The putative sk-coding DNA fragment was amplified from* S. pyogenes* DT7 genomic DNA by PCR with* Taq* DNA polymerase. Based on the nucleotide sequence of the* sk* gene from* S. pyogenes* strain (GenBank: Z48617), 3 oligonucleotides, mSKF GGC GGATCC CATATG ATTGCTGGACCTG, and SKF: GCC CAT GGG CAA AAA TTA CTT AT and SKR GCC TCG AGT TTG TCB TTA GGG TT were designed as primers for introduction of the underlined* Bam*HI and* Xho*I restriction sites, respectively. The PCR mixture contained 2.5 *μ*L 10x PCR buffer; 2 *μ*L of 2.5 mM dNTP; 2.5 *μ*L of 25 mM MgCl_2_; 0.5 *μ*L genomic DNA (50–100 ng); 0.25 *μ*L 5 unit* Taq* polymerase, and 1 *μ*L each primer (10 pmol), supplemented with 15.25 *μ*L distillated water to a final volume of 25 *μ*L. The thermocycler conditions were as follows: 95°C/4′; 30 cycles of (95°C/30′′, 52°C/45′′, 72°C/45′′); 72°C/10′. The PCR products amplified from the genomic DNA with the primer pair SKF and SKR were inserted into the cloning vector pJET1.2/blunt, resulting in pJSK. DNA sequencing was performed on ABI PRISM 3100 Avant Genetic Analyzer. Sequence alignments were constructed and analyzed using the program MegAlign DNAStar. It was followed by ligation of the* BamHI-Xho*I digested PCR products (with the primer pair mSKF and SKR) with pET22b+ linearized by the same enzymes, resulting in pESK under the control of the T7-promoter induced by isopropyl-*β*-D-thiogalactopyranoside (IPTG) and possessing the ampicillin marker. The streptokinase rSKhis encoded by the plasmid pESK contains the mature streptokinase fused with the 6x histidine-tag and no native leader sequence.

### 2.5. rSK Expression

The transformant* E. coli* BL21/pESK was cultivated overnight in 5 mL of LB medium containing 5 *μ*L of 100 mg/mL ampicillin at 37°C on an orbital shaker at 200 rpm. Overnight culture (2 mL) was inoculated in a 1-liter Erlenmeyer flask containing 200 mL of LB broth and 200 *μ*L of 100 mg/mL ampicillin. The culture was grown at 37°C with agitation at 200 rpm and until an optical density (OD) at 600 nm reached 0.6 (for approximately 2.5 h); then 200 *μ*L of 100 mM IPTG was added. The culture was continuously incubated at 37°C with agitation at 200 rpm for 3–6 h induction. Cells were harvested by centrifugation at 6000 rpm for 10 min at 4°C. Wet weight cells were used for protein purification.

### 2.6. Purification of Streptokinase

The fusion form rSKhis carrying a C-terminal 6xHis tag was expressed in* E. coli *BL21. To purify rSK, 100 mg wet weight cells from a 120 mL culture in LB medium were harvested by centrifugation and suspended in 10 mL of guanidine lysis buffer containing 6 M guanidine hydrochloride, 20 mM sodium phosphate, 500 mM NaCl, and pH 7.8. The cell suspension was sonificated (three bursts of 1 min each at 1 min interval). After 30–60 min incubation in ice with slight shaking, the cell lysate was centrifuged at 13000 rpm and 4°C for 25 min to remove cell debris. A volume of 8 mL cell lysate was applied to a Ni-NTA column (Invitrogen Corp., Carlsbad, USA) containing 2 mL resin which was equilibrated with denaturing binding buffer and incubated for 45 min at room temperature with gentle hand shaking for several times. The column was washed with 4 times of 8 mL denaturing wash buffer. The bound protein was eluated with 8 mL of denaturing eluation buffer. Then 6 mL of the enzyme extract was applied to a Bio-gel column (2,6 × 6 cm) with elution of 50 mM Tris-HCl buffer (pH 8) at a flow rate of 25 mL/h and then washed with the same buffer.

### 2.7. Streptokinase Renaturation

The pool of purified SK fragments were renaturated using 50 mM phosphate buffer pH 7 supplemented with 10% (w/v) glycerol and different detergents (0.5% (w/v) Triton X-100, 1% (w/v) Tween 20, 1.5% (w/v) Tween 80) [11]. Diluted cell lysate (1 : 200) and purified rSK (1 : 100) in renaturation buffer were incubated at 37°C for 1 h and 4°C for 6 h. The residual activity was then determined as described below.

### 2.8. Streptokinase Assay

To estimate the activity of the purified rSK, 10 *μ*L purified protein solution was added to 10 *μ*L of 50 mM Tris buffer pH 7.5 containing 0.05 unit of human plasminogen and incubated at 37°C for 30 min. The color reaction was developed by the addition of 40 *μ*L of 1 mM AAS solution and incubated at 37°C for 15 min. The reaction was stopped by the addition of 10 *μ*L of 0.4 N acetic acid. The absorbance was read at 405 nm against a blank containing human plasminogen, Tris buffer, and AAS but without rSK solution. The activity was estimated using standard SK (Sigma Aldrich Co., St. Luis, USA). One unit (U) of rSK was defined as one unit of standard SK, which liquefies a standard clot of fibrinogen, plasminogen, and thrombin at 37°C and pH 7.5 for 10 min.

### 2.9. Protein Electrophoresis and Quantification

The homogeneity and molecular mass of the streptokinase were determined by 12.5% SDS polyacrylamide gel electrophoresis [16] with Biometra equipment (Göttingen, Germany). Proteins were visualized by staining with Coomassie Brilliant Blue R-250 or with 0.1% (w/v) of silver nitrate. Protein concentrations were measured by Bradford assay with the bovine serum albumin as standard [17].

### 2.10. MALDI-TOF Mass Spectrometry

The rSK was identified by MALDI-TOF mass spectrometry as previously described [18]. The predicted protein band on SDS-PAGE was cut out and the target protein was digested by trypsin treatment into small peptide fragments. The mixture of peptides was analyzed on nano-LC liquid chromatography and ionized by the ESI (electrospray ionization). The mass spectra were obtained by QSTAR XL mass spectrometer (Applied Biosystems, MDS SCIEX, Canada) with a nano-ESI ion source. Protein fragments were identified by the Mascot v1.8 Search Software from the database (NCBInr, SwissProt). Peptide fragments showing ion scores above 42 were identified uniquely or high-similarly with *P* < 0.05.

### 2.11. Biochemical Characterization of rSK

The pH and temperature optimum of rSK were determined by measuring the activity as described above using 100 mM potassium phosphate buffer (pH 5.5–7.5) and 100 mM Tris-HCl buffer (pH 7.5–10) at 37°C, and in the temperature range of 4 to 60°C using 100 mM potassium phosphate buffer, pH 7.5, respectively.

For the determination of temperature and pH stability, the purified rSK, 0.1 *μ*g for each reaction, was preincubated in 100 mM potassium phosphate buffer pH 7 at different temperatures 4–60°C for 0–96 h, and pH range from 4 to 9.5 (pH 4-5, 100 mM potassium acetate buffer; pH 5.5–7.5, 100 mM potassium phosphate buffer; and pH 7.5–9.5, 100 mM Tris-HCl) at 37°C for 0–48 h, respectively. The residual activity was then determined.

Effect of surfactants on the activity of rSK was check by mixture of 0.4 unit purified rSK and substrate and supplemented with either Triton X-100, Tween 20, or Tween 80, each at a final concentration of 0.5, 1.0, 1.5, and 2.0% (w/v) in appropriate buffer pH 7 and incubated at 37°C for 60 min. The residual activity of rSK was determined as described above.

The effect of additives on the activity of the purified rSK was investigated by incubating the mixture of 0.4 unit of the purified rSK and either of Ag^+^, Ca^2+^, Co^2+^, Cu^2+^, Fe^2+^, K^+^, Mn^2+^, Ni^2+^, Zn^2+^, or EDTA, at a final concentration of 1, 3, and 5 mM. The reaction mixtures were incubated at 28°C for 60 min. The residual activity of rSK was then measured as shown above. All measurements were carried out in triplicate with the resulting values being the mean of the cumulative data obtained.

## 3. Results and Discussion

### 3.1. Gene Cloning and Analysis

The recombinant plasmid pTSK with inserted* sk* gene was sequenced and aligned with sequences from GenBank using DNAstar. Nucleotide sequence of* sk* gene from* S. pyogenes *DT7 exhibited 84.4% to 99.6% identities with sequences from* Streptococcus pyogenes *groups of A, C, and G strains in GenBank (CP000262, CP000261, M19347, AM903378, and AY234136). The putative amino acid sequence of the gene* sk* showed 77.9 to 99.3% identities with the corresponding amino acid sequences from the abovementioned* Streptococcus pyogenes* strains. The sequence was deposited in the GenBank with an accession number of ACG50170.

### 3.2. Expression and Purification of SK

**Figure 1:**
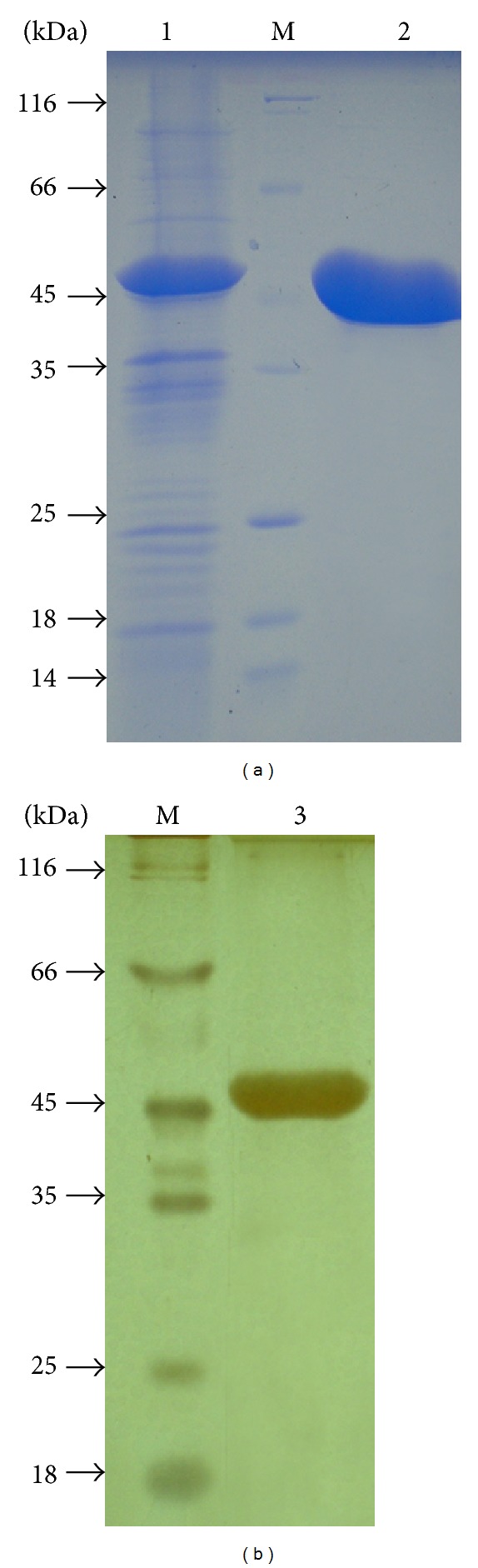
SDS-PAGE of the purified rSK by ProBond Resin. Lane 1,* E. coli* BL21/pESK cell lysate; Lane 2, purified rSK stained by using Coomassie Brilliant Blue R250; Lane 3, purified rSK stained by using silver nitrate; Lane M, molecular standards indicated in kDa.

The DNA fragment (1245 bps) encoding the mature streptokinase (SK) truncated 26 N-terminal amino acids from* S. pyogenes *DT7 was inserted into pET22b+ vector at the* Bam*HI and* Xho*I sites resulting in the recombinant plasmid pESK. The transformant* E. coli* BL21/pESK was grown in LB medium for the SK production. After IPTG induction, the cells were collected and used for purification and renaturation. The expression level of rSK as inclusion bodies by* E. coli* BL21/pESK was 60% of the total proteins (Figure 1(a), lane 1) using Dolphin 1D software. This level was as high as that (65%) reported by Zhang et al. (1999) [19] and more than two to four times as high as those (25%) reported by [20], (20%) by [21], and 15% by [22].

### 3.3. Renaturation of Streptokinase

The cell lysate was renatured by using various surfactants including Triton X-100, Tween 20, and Tween 80 each or in combination with glycerol. Triton X-100 was known as detergent to dissolves and refolding aggregated protein. In absence of surfactants, rSK exhibited the same activity (182–189 U/mL) with or without glycerol (Table 1). The addition of surfactants increased the rSK activity obviously to 3.5–3.8 folds without glycerol, but steeply to 25.2–30.6-folds in combination with glycerol at 37°C for 60 min, even to 36.1–41.7 folds at 4°C for 6 h. At lower temperature (4°C), the enzyme activity was recovered better than at higher temperature (37°C), increased by 26–43%. The combination of glycerol at the concentration of 10% (w/v) and Triton X-100 at the concentration of 0.5% (w/v) recovered the highest activity of rSK and reached 7,591 U/mL at 4°C (Table 1). The renaturation of the purified rSK with 10% glycerol containing 0.5% Triton X-100 at 4°C for 6 h and at 37°C for 1 h recovered the enzyme activity of 28.6 and 36.5 folds, respectively (Table 2), corresponding to the specific activity of 10,312.5, and 11,264.2 U/mg protein. The reason the renaturation efficiency in this study was much higher than that reported by Cherish Babu et al. (2008). At the same conditions for treatment, the enzyme activity was recovered with only 9.7 folds in comparison to control [11].

### 3.4. Purification of Recombinant SK

rSK from* S. pyogenes* DT7 overexpressed by* E. coli* BL21/pESK cells was purified through affinity chromatography column of Ni^2+^-ProBond resin to the homogeneity on SDS-PAGE with a molecular mass of approximately 47 kDa (Figure 1, lane 2). The purified rSak gained a specific activity of 10,336 U/mg proteins with a purification factor of 2.56 and a yield of 52% (Table 3). The solution containing rSK protein was loaded onto Biogel P-100 packed column for fractionating and obtained with a purity of 95.7% and specific activity of 11,558 U/mg (Figure 1, lane 3).

### 3.5. Identification of Recombinant SK

**Figure 2:**
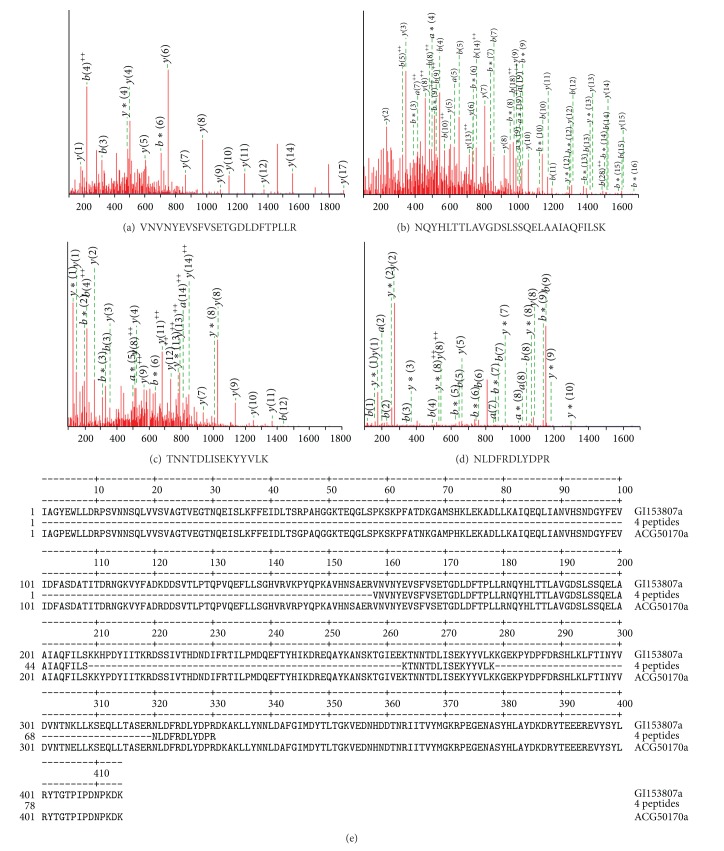
Monoisotopic mass of three neutral identified peptides. (a) VNVNYEVSFVSETGDLDFTPLLR position 158–180 (a); (b) NQYHLTTLAVGDSLSSQELAAIAQFILSK position 181–208; (c) TNNTDLISEKYYVLK position 263–279; (d) NLDFRDLYDPR position 320–330 found in gi: 153807, streptokinase from* Streptococcus pyogenes* (GenBank, AAA26973) corresponding to ion scores of 102, 111, 73, and 51 with *P* < 0.05, respectively. (e) Alignment of four neutral identified peptides (4 peptides) with streptokinase from* Streptococcus pyogenes* AAA26973 (gi153807) and rSK from* S. pyogenes* DT07 (ACG50170).

The single protein on SDS-PAGE (Figure 1, lane 3) was cut out from the gel and used for LC-ESI-MS/MS analysis of mass spectrum database by using Mascot v1.8 program. The total score of SK identification was 203 to 509 and matched peptides were 29 to 39 fragments. Four peptide fragments of the purified enzyme identified by MALDI-TOF mass spectrometry agreed with those of the streptokinase found in GenBank gi|153807, streptokinase (*S. pyogenes*) VNVNYEVSFVSETGDLDFTPLLR (position 158–180) (Figure 2(a)), NQYHLTTLAVGDSLSSQELAAIAQFILSK (position 181–209) (Figure 2(b)), TNNTDLISEKYYVLK (position 263–278) (Figure 2(c)), NLDFRDLYDPR (position 320–330) (Figure 2(d)), corresponding to a monoisotopic mass of 2613.3, 3117.63, 1799.93, and 1422.69 Da and to m/z ion scores of 102, 111, 73, and 51, respectively. Whereas the peptide fragments showing ion scores above 42 were identified uniquely or highly similarly to *P* < 0.05. These peptides of the recombinant streptokinase expressed by* E. coli*/pESK showed 100% identity with the corresponding fragments of the putative streptokinase protein from* S. pyogenes* (gi|153807) (Figure 2(e)).

### 3.6. Temperature and pH Optimum

**Figure 3:**
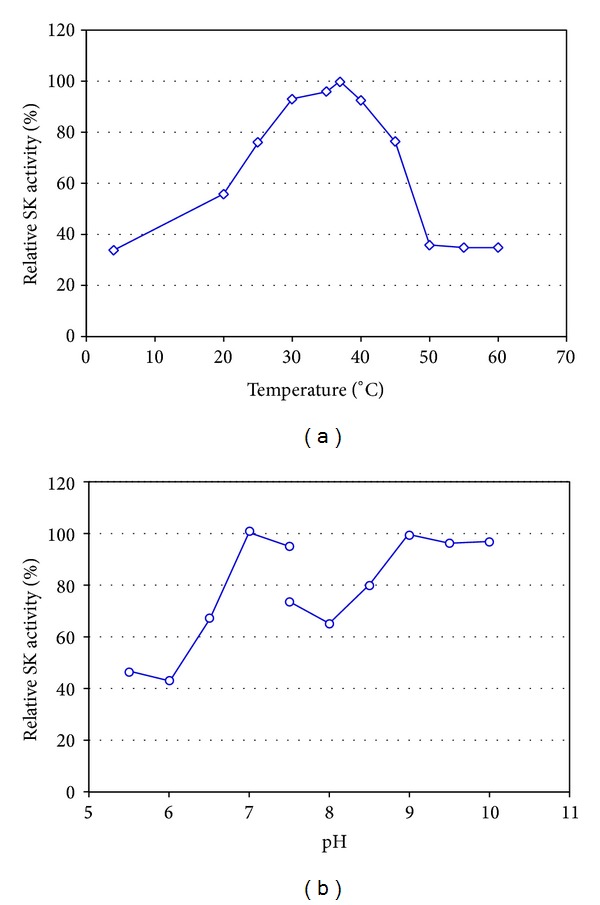
Temperature (a) and pH (b) optimum of rSK from* S. pyogenes* DT07.

The temperature and pH optimum for the reaction of SK-plasmin were observed at 37°C and pH 7 (Figures 3(a) and 3(b)). The enzyme showed over 80% activity at the temperature range from 25 to 45°C and pH 6.7–7.5 (for 100 mM potassium phosphate buffer) and pH 8.5–10 (for Tris-HCl buffer) in comparison with the optimum activity. The temperature optimum for the SK-plasmin reaction was in agreement with that from other reports. Rajagopalan et al. (1987) reported that the reactions of *α*2-macroglobulin (*α*2M) with plasmin or streptokinase-plasmin (ogen) (SkP1) was markedly temperature-dependent and initial rates of reaction at 0 and 24°C were only 3 and 40% of the rate of 37°C, respectively [23]. Mumme et al. (1993) reported that the highest fibrinolysis activity with streptokinase was obtained at 40°C, with lower activities having been recorded at both higher and lower temperatures [24]. The optimum temperature and pH of streptokinase from *β*-haemolytic streptococci were 27–37°C and 7 [25]. Another thrombolytic agent, closely related to the streptokinase, staphylokinase (Sak) from* Staphylococcus aureus* exhibited the same profile. The native Sak from* S. aureus* V8 showed the pH optimum at pH 7.5 and 8.5 [26]. The temperature and pH optimum for Sak from* S. aureus* QT08 expressed in* E. coli* and* P. pastoris* were observed at 30–37°C, pH 7, and pH 9 [27] and pH 7,5, and pH 8.5 [28], respectively.

Why the streptokinases and staphylokinases shared a common property that the optimum temperature was not more than 40°C and pH optimum exhibited 2 peaks? because the fibrinolytic activity of streptokinase originates in its ability to activate blood plasminogen to plasmin, the enzyme that degrades fibrin cloth through its specific lysine binding site [29]. The temperature optimum for the human plasmin was at 37°C [30] and the optimal pH value for the human plasmin and that for SK or Sak were significantly different.

### 3.7. Temperature and pH Stability

**Figure 4:**
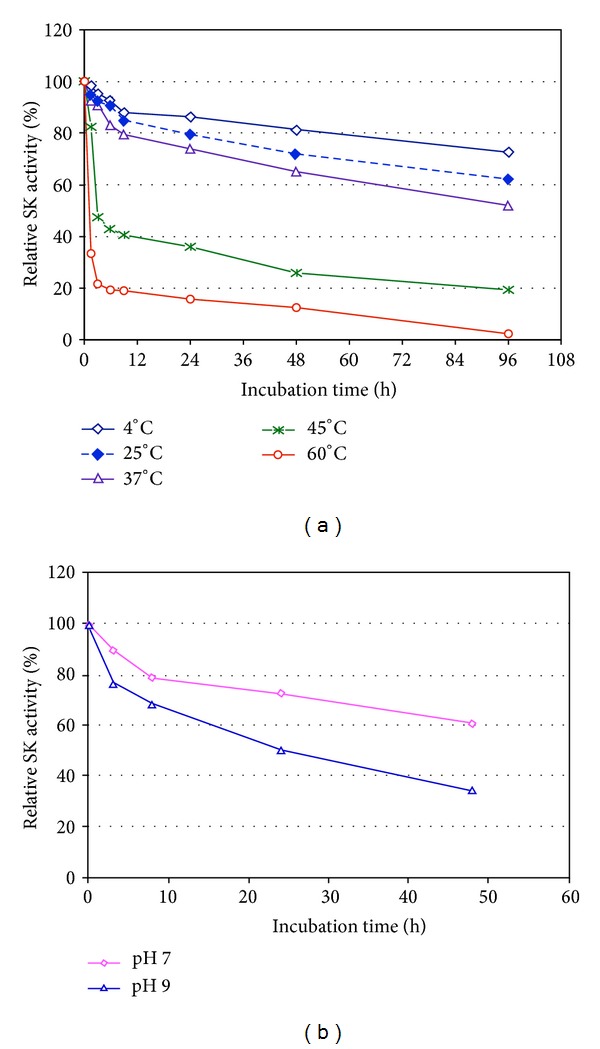
Temperature (a) and pH (b) stability of rSK from* S. pyogenes* DT07.

The streptokinase from* S. pyogenes* DT7 was stable up to 37°C and retained more than 80% of its initial activity after incubation for 9 h and more than 50% after incubation for 96 h (Figure 4(a)). The enzyme exhibited more stability at pH 7 than at pH 9 and retained more than 73% of its initial activity after incubation at pH 7 for 24 h, whereas it retained only more than 65% of its initial activity after incubation at pH 9 for 8 h (Figure 4(b)). K. Vesterberg and O. Vesterberg (1972) also reported that the concentrated material containing Sak from* S. aureus* V8 was stable at refrigerator temperature over a pH range of 3.0–8.5. Sak from* S. aureus* QT08 expressed in* E. coli* and* P. pastoris* was stable at a temperature range from 25°C to 50°C, and at a pH range from 7 to 9 after incubation for 2 h with a residual activity of more than 70% [26, 28]. The results depicted in Figure 4(b) indicating that there were two sharp peak, one at pH 7.0 and the other one at pH 9.0 with the activity of 100% and 98%, respectively. The experiments of the optimal pH value for the high level activity of rSK were rather complicated since two reactions happened continuously in the same reaction mixture: at first, the activation reaction of plasminogen to plasmin was activated by rSK, and second, the digestion process of AAS was catalyzed by plasmin. The optimal pH value for human plasmin and that for SK was significantly different; therefore, this could cause the appearance of second peak activity. The data depicted in Figure 4(b) showing that the second peak activity at pH value of 9,0 might therefore be due to optimal pH for the plasmin activity in Tris-HCl buffer. Similarly, these observations were also reported by K. Vesterberg and O. Vesterberg (1972) in which staphylokinase was a plasminogen activator.

### 3.8. Effect of Surfactants

The addition of either Tween 80, Tween 20, or Triton X-100 at the final concentration of 0.5–2% (w/v) in reaction mixture showed an activation of the streptokinase from* S. pyogenes* DT07 up to 150% of its original activity. The enzyme activity increased up to 154% after incubation for 24 h but deeply decreased to 18% after longer incubation for 48 h (Table 4). Similarly, Cherish Babu et al. (2008) reported that rSK was treated with guanidine and then supplemented with Triton X-100 that enhanced the activity of rSK.

### 3.9. Effect of Metal Ions and EDTA

In the present study, effect of various additives on the purified rSK activity was investigated. The addition of EDTA and metal ions showed a clear effect on the streptokinase activity. EDTA, Mn^2+^, and K^+^ inhibited the enzyme partially whereas Ag^+^, Ca^2+^, and Co^2+^ exhibited a strong inhibition. But the metal ions Cu^2+^, Fe^2+^, Ni^2+^, and Zn^2+^ at a final concentration of 1 mM completely inhibited the streptokinase (Table 5). In previous studies, it was also observed that the addition of Zn^2+^ and Cu^2+^ almost completely inhibited the activity of the recombinant staphylokinase from* Staphylococcus aureus* QT08 [27] and the native staphylokinase from* S. aureus* V8 [31], another thrombolytic agent, closely related to the streptokinase. Why the streptokinases and staphylokinases shared a common property that addition of Zn^2+^ and Cu^2+^ resulted in almost completely inhibition of activities? Because the plasmin completely lost its activity when it was incubated with Zn^2+^ and Cu^2+^ [32, 33].

## 4. Conclusion

SK is a promising blood-clot dissolving agent for the treatment of patients suffering from a heart attack. It would be desirable to produce high yield of protein with high activity for thrombolytic therapy. In the present study, a* sk* gene from* Streptococcus pyogenes *DT7 was overexpressed in* E. coli *with a level of 60% of total proteins which is highest yield of any rSK expressed in* E. coli* till date. A simple renaturation system dramatically covered the rSK activity with 41 folds, which was not reported before. Overproduction of rSK in* E. coli* in combination with a simple and highly effective renaturation made the recombinant* E. coli* become a potential strain for industrial SK production.

## Figures and Tables

**Table 1:** Effect of surfactant, glycerol, and temperature on the renaturation of cell lysate *E. coli*/pESK.

Parameter	SK activity (U/mL)		
	at 37°C for 1 h	at 37°C for 1 h + 10% glycerol	at 4°C for 6 h + 10% glycerol
0.5% Triton X-100	643.4 ± 3.6	5574.0 ± 36.2	7591.3 ± 45.2
1% Tween 20	657.6 ± 5.7	4592.8 ± 27.1	6587.7 ± 27.1
1.5% Tween 80	691.6 ± 6.6	5226.8 ± 50.6	6578.7 ± 63.3
No surfactant	182.1 ± 1.8	184.3 ± 3.6	189.4 ± 3.6

**Table 2:** The renaturation of purified rRSK.

Fraction number	rSK activity (U/mg)		
	at 37°C for 1 h	at 4°C for 6 h + 0.5% Triton X-100 and 10% glycerol	at 37°C for 1 h + 0.5% Triton X-100 and 10% glycerol
1	69.1 ± 1.0	1622.4 ± 29.3	2519.0 ± 15.7
2	360.5 ± 4.8	10312.5 ± 55.2	11264.2 ± 27.6
3	199.9 ± 1.4	2039.3 ± 21.1	3862.5 ± 15.2
4	55.3 ± −0.3	1516.9 ± 18.9	1907.6 ± 11.8

**Table 3:** Purification steps of the streptokinase from *E. coli*/pESK.

Total proteins	Purified proteins	Specific activity (U/mg) of		Yield (%)	Purification factor
		supernatant of cell lysate	purified rSK		
90375 mg	21789 mg	4568	10336	52%	2.26

**Table 4:** Effect of surfactants on streptokinase activity.

Detergent	Residual activity (%) at the concentration (%)			
	0.5	1.0	1.5	2.0
Tween 20	137.5 ± 2.6	138.3 ± 1.8	135.0 ± 2.3	136.5 ± 2.6
Tween 80	119.1 ± 2.8	125.9 ± 2.6	142.2 ± 1.5	115.4 ± 2.2
Triton X-100	150.3 ± 1.9	140.6 ± 3.6	143.6 ± 0.9	126.6 ± 2.0

	Relative activity (%) after incubation for (h)			
	0	8	24	48

Tween 20	117.6 ± 1.5	149.2 ± 1.3	154.7 ± 1.2	21.9 ± 0.2
Tween 80	121.9 ± 1.9	151.9 ± 0.7	154.4 ± 1.1	27.6 ± 0.2
Triton X-100	119.3 ± 1.6	162.6 ± 1.3	15.4 ± 0.1	18.1 ± 0.2

**Table 5:** Effect of metal ions on streptokinase activity.

Additive	Residual activity (%) at the concentration (mM)		
	1	3	5
AgNO_2_	16.1 ± 0.1	n.d.	n.d.
CaCl_2_	14.5 ± 0.2	n.d.	n.d.
CoCl_2_	15.6 ± 0.1	n.d.	n.d.
EDTA	50.4 ± 0.2	70.5 ± 0.3	88.2 ± 0.4
KCl	18.5 ± 0.1	25.2 ± 0.2	59.6 ± 0.5
MnSO_4_	40.5 ± 0.4	54.2 ± 0.3	68.4 ± 0.5
